# Dental Anxiety and Oral Health-Related Quality of Life Among Adults in the United Arab Emirates: A Cross-Sectional Study

**DOI:** 10.3390/healthcare14020219

**Published:** 2026-01-15

**Authors:** Nada Tawfig Hashim, Muhammed Mustahsen Rahman, Riham Mohammed, Md Sofiqul Islam, Vivek Padmanabhan, Sharifa Jameel Hossain, Nallan C. S. K. Chaitanya, Noran Osama Mohammed, Asawer Ahmed Saeed, Shahista Parveen Dasnadi

**Affiliations:** 1 Department of Periodontics, RAK College of Dental Sciences, RAK Medical & Health Sciences University, Ras-AlKhaimah 12973, United Arab Emirates; mustahsen@rakmhsu.ac.ae; 2 Department of Oral Surgery, RAK College of Dental Sciences, RAK Medical & Health Sciences University, Ras-AlKhaimah 12973, United Arab Emirates; riham.abdelraouf@rakmhsu.ac.ae; 3 Department of Operative Dentistry, RAK College of Dental Sciences, RAK Medical and Health Sciences University, Ras-AlKhaimah 12973, United Arab Emirates; sofiqul.islam@rakmhsu.ac.ae; 4 Department of Pediatric and Preventive Dentistry, RAK College of Dental Sciences, RAK Medical & Health Sciences University, Ras-AlKhaimah 12973, United Arab Emirates; vivek.padmanabhan@rakmhsu.ac.ae; 5 RAK College of Dental Sciences, RAK Medical & Health Sciences University, Ras-AlKhaimah 12973, United Arab Emirates; sharifa.jameel@rakcods.com (S.J.H.); noran.20902056@rakmhsu.ac.ae (N.O.M.); asawer.20902028@rakmhsu.ac.ae (A.A.S.); 6 Department of Oral Medicine and Radiology, RAK College of Dental Sciences, RAK Medical & Health Sciences University, Ras-AlKhaimah 12973, United Arab Emirates; krishna.chytanya@rakmhsu.ac.ae; 7 Department of Orthodontics, RAK College of Dental, RAK Medical & Health Sciences University, Ras-AlKhaimah 12973, United Arab Emirates; shahistha.parveen@rakmhsu.ac.ae

**Keywords:** dental anxiety, oral health-related quality of life, OHIP-14, MDAS, oral health behavior, psychosocial determinants, UAE, cross-sectional study

## Abstract

**Background**: Dental anxiety is a common psychological condition that may influence patients’ perceptions of oral health and well-being. Although its association with oral health-related quality of life (OHRQoL) has been widely studied internationally, evidence from the United Arab Emirates (UAE) remains limited. **Objectives**: This study aimed to examine the association between dental anxiety and OHRQoL among adult patients attending an academic dental clinic in the UAE. **Methods:** A cross-sectional study was conducted among adult dental patients using a non-probabilistic sampling approach. Dental anxiety was assessed using the Modified Dental Anxiety Scale (MDAS), and OHRQoL was measured using the Oral Health Impact Profile-14 (OHIP-14). Descriptive statistics and nonparametric tests were used for bivariate analyses. Multiple linear regression was applied as an exploratory approach to assess adjusted associations between dental anxiety and OHRQoL after accounting for age and gender. **Results:** Higher dental anxiety scores were independently associated with poorer OHRQoL after adjustment for age and gender. Bivariate analyses showed no statistically significant differences in dental anxiety or OHRQoL scores between men and women; however, subgroup comparisons should be interpreted cautiously given the sample size. The findings indicate a consistent association between higher anxiety levels and greater perceived oral health impact within the study population. **Conclusions**: Dental anxiety was associated with impaired oral health-related quality of life among adult dental clinic attendees in the UAE. These findings reflect associations observed within a modest, non-probabilistic, cross-sectional sample and should not be interpreted as causal or generalized to the wider population. Further longitudinal and population-based studies incorporating clinical and contextual variables are needed to clarify temporal relationships and strengthen external validity.

## 1. Introduction

Understanding dental anxiety as a major psychological determinant of oral health has gained increasing prominence in contemporary dental public health research [1]. Dental anxiety, characterized by excessive fear, apprehension, or stress related to dental treatment, affects approximately 10–20% of the global population and represents one of the most prevalent specific anxieties worldwide [2]. Individuals with heightened dental anxiety frequently delay or avoid dental appointments, resulting in the progression of untreated caries and periodontal disease, functional impairments, and diminished quality of life. Thus, dental anxiety is not only an emotional burden but also a behavioral barrier that perpetuates poor oral health outcomes across the lifespan [3,4].

Parallel to the rising recognition of dental anxiety, oral health-related quality of life (OHRQoL) has emerged as a central patient-reported outcome in modern dentistry [5]. OHRQoL captures the broader functional, psychological, and social impacts of oral conditions, reflecting elements such as pain, difficulty in chewing, impaired speech, social withdrawal, and reduced self-esteem [6]. The Oral Health Impact Profile (OHIP-14), a widely validated instrument, measures these multidimensional outcomes and has been used globally to quantify the subjective burden of oral disease beyond clinical indicators alone [7]. Evidence consistently demonstrates that poor oral health significantly impairs daily performance, emotional stability, and social behavior, highlighting the need to assess quality of life as a core component of oral healthcare delivery [8,9].

A growing body of research has identified a strong association between dental anxiety and OHRQoL [3,10]. Individuals with higher anxiety levels often experience greater oral health impairment due to care avoidance, lower tolerance for dental discomfort, and increased psychological distress during or after dental treatment. Studies from Europe, Asia, and North America consistently report that dental anxiety is negatively correlated with OHRQoL, with anxious individuals exhibiting higher OHIP-14 scores, reflecting greater functional limitations and psychosocial burden [11,12]. This relationship creates a cyclical pattern: dental anxiety leads to poor oral health, which further worsens OHRQoL, reinforcing the underlying fear and anxiety associated with dental care.

Despite the well-documented global burden of dental anxiety, evidence from the Middle East—particularly the United Arab Emirates (UAE)—remains limited and fragmented [13,14]. Cultural norms, healthcare-seeking behaviors, social expectations, and patterns of dental service utilization are likely to shape both the experience of dental anxiety and its perceived impact on daily functioning and quality of life in this region [15]. Importantly, the UAE has undergone rapid transformation in oral healthcare delivery, with expanded access to dental services, preventive programs, and patient-centered care models; however, psychological barriers to care, including dental anxiety, continue to be reported [15]. Existing regional studies suggest demographic variability in anxiety levels [16], yet comprehensive and contemporaneous investigations linking dental anxiety with oral health-related quality of life are scarce. Moreover, few studies within the UAE context have simultaneously assessed these constructs using validated instruments such as the Modified Dental Anxiety Scale (MDAS) and the OHIP-14, limiting the ability to contextualize international evidence and to inform locally relevant clinical and educational strategies. Given these gaps, investigating the association between dental anxiety and OHRQoL in the UAE is both timely and crucial. Such evidence can help clinicians identify at-risk groups, develop targeted anxiety-reduction strategies, improve patient satisfaction, and ultimately enhance oral health outcomes. Moreover, understanding how demographic variables such as age, gender, and dental attendance patterns influence this relationship can inform the design of culturally responsive interventions and public health policies.

Conceptually, the observed association between dental anxiety and oral health-related quality of life can be viewed within established behavioral frameworks such as fear-avoidance and social learning models, which propose that anxiety-related perceptions and expectations may shape health behaviors and subjective health experiences without presupposing causal direction [11,12].

Therefore, the present study aimed to examine the association between dental anxiety and oral health-related quality of life (OHRQoL) among adult dental patients in the United Arab Emirates and to explore how this association varies according to selected demographic characteristics. Given the cross-sectional design, the study was intended to generate associational evidence rather than causal inference.

## 2. Materials and Methods

### 2.1. Study Design

A cross-sectional analytical study was conducted to examine the association between dental anxiety and oral health-related quality of life (OHRQoL) among adult dental patients. This design enabled the assessment of anxiety levels, perceived oral health impact, and their co-occurrence at a single point in time, in line with established epidemiological approaches.

The study was intentionally centered on patient-reported outcomes, focusing on subjective experiences of dental anxiety and perceived oral health impact rather than on objective clinical oral health indicators. As such, standardized clinical assessments of caries experience, periodontal status, malocclusion, or tooth loss were not collected. Accordingly, the analytical models were structured to explore psychosocial associations rather than to estimate the full spectrum of biological or clinical determinants of OHRQoL.

### 2.2. Study Setting and Population

The study was carried out at RAK College of Dental Sciences (RAKCODS), Ras Al Khaimah, United Arab Emirates. The setting serves a diverse adult population and provides a representative clinical environment for evaluating psychosocial factors related to dental care. All data were collected between November 2024 and April 2025. The study population included adult patients aged 18 years and above who visited the dental outpatient clinics during the study period.

### 2.3. Inclusion and Exclusion Criteria

Inclusion criteria comprised adults aged 18 years or older who were able to comprehend and independently complete the study questionnaires in either Arabic or English and who provided written informed consent.

Participants were excluded if they had any cognitive impairment, intellectual disability, or psychiatric condition that, in the judgment of the investigators, interfered with their ability to understand or reliably complete the questionnaires. Individuals requiring assistance to complete the questionnaires were not enrolled.

These criteria were applied uniformly during recruitment to ensure consistency in participant selection and to minimize information bias related to self-reported measures.

### 2.4. Sampling Strategy and Sample Size

A purposive sampling approach was employed to recruit eligible participants attending the dental clinics. This approach ensured targeted recruitment of individuals who met the inclusion criteria and were representative of the local patient population.

The minimum required sample size was calculated using Cochran’s formula for cross-sectional studies:

n = (Z^2^ × P × (1 − P))/d^2^

where

- n = required sample size
- Z = Z-value for the desired confidence level (1.96 for 95% CI)
- P = estimated proportion of the population (0.5 used for maximum variability)
- d = margin of error (0.05)

The estimated minimum sample size was 385 participants. To account for a potential 15% non-response rate, the adjusted target was 450 participants. A total of 138 complete responses were obtained and included in the final analysis.

Although the a priori sample size calculation indicated a minimum requirement of 385 participants, the final analytical sample comprised 138 complete responses. This discrepancy reflects practical constraints related to recruitment duration and response rates commonly encountered in questionnaire-based clinical studies. Importantly, the achieved sample size is not presented as evidence of design adequacy, precision, or inferential power.

The original sample size calculation was undertaken to estimate population proportions in a cross-sectional context and was not intended to support hypothesis-confirming association testing or multivariable regression modeling. Accordingly, all inferential analyses reported in this study were conducted explicitly as exploratory and associational, and any statistical significance observed is interpreted descriptively rather than as confirmation that the study was powered to detect or exclude specific effects. The findings should therefore be regarded as hypothesis-generating.

### 2.5. Data Collection Instruments

Two validated, self-administered Arabic and English questionnaires were used.

#### 2.5.1. Modified Dental Anxiety Scale (MDAS)

Dental anxiety was measured using the Modified Dental Anxiety Scale, a 5-item tool assessing fear across different dental situations. Each item is rated on a 5-point Likert scale (1 = not anxious; 5 = extremely anxious). Total scores range from 5 to 25, categorized as:

- Not anxious: 5–9
- Slightly anxious: 10–12
- Fairly anxious: 13–17
- Very anxious: 18–20
- Extremely anxious: 21–25

The MDAS has demonstrated strong reliability in Middle Eastern populations.

#### 2.5.2. Oral Health Impact Profile (OHIP-14)

OHRQoL was assessed using the OHIP-14, which includes 14 items across seven domains (functional limitation, physical pain, psychological discomfort, physical disability, psychological disability, social disability, and handicap). Items are scored on a 5-point scale (0 = never; 4 = very often). Higher total scores indicate worse OHRQoL.

### 2.6. Data Collection Procedure

Participants were approached in the waiting area of the dental clinics. After providing informed consent, they completed both questionnaires anonymously. Trained research assistants supervised the process and offered clarification as needed, without influencing responses.

All completed forms were reviewed for completeness before data entry into an encrypted database.

### 2.7. Ethical Approval

Ethical approval was obtained from the Research and Ethics Committee of RAK Medical and Health Sciences University (Approval No.: RAKMHSU-HEC-51-2023/25-UG-D). Participation was voluntary, all information was anonymized, and no personal identifiers were collected. The study adhered to the principles of the Declaration of Helsinki.

### 2.8. Statistical Analysis

Data were analyzed using SPSS version 29, with statistical significance set at *p* < 0.05. The analytical approach was explicitly exploratory and associational. Multivariable regression modeling was used to estimate adjusted associations between dental anxiety and oral health-related quality of life (OHRQoL) rather than to infer causality. Covariates (age and gender) were selected a priori based on consistent reporting in the literature and availability in the dataset. The model was not intended to represent a comprehensive causal framework, and residual confounding by unmeasured variables—such as socioeconomic status, oral hygiene behaviors, psychological comorbidities, or dental service utilization—cannot be excluded. No formal power calculation was performed for the association or regression analyses, and the resulting estimates should therefore be interpreted with caution with respect to precision and generalizability. Prior to interpretation, standard diagnostic checks were undertaken to assess the suitability of the multiple linear regression model. This included evaluation of residual distribution, variance consistency, and inspection for influential observations. In addition, the independent variables included in the model were examined for redundancy and excessive interdependence, with no indications of problematic collinearity observed. On this basis, the regression model was considered appropriate for exploratory associational analysis within the constraints of the study design.

#### 2.8.1. Assessment of Normality

Normality of MDAS and OHIP-14 total scores was evaluated using the Shapiro–Wilk test. Both variables demonstrated significant deviation from normality (MDAS: W = 0.90, *p* < 0.001; OHIP-14: W = 0.93, *p* < 0.001). Therefore, non-parametric tests were used when appropriate.

#### 2.8.2. Descriptive Statistics

Descriptive statistics included frequencies, percentages, means, medians, standard deviations, and interquartile ranges. Anxiety categories and OHRQoL impact levels were assigned based on established scoring guidelines.

#### 2.8.3. Inferential Statistics

Mann–Whitney U test was used to compare MDAS and OHIP-14 scores by gender.

Kruskal–Wallis test (recommended over ANOVA for non-normal data) was used to compare OHRQoL across anxiety categories, followed by post hoc pairwise comparisons with Bonferroni correction.

Spearman’s rank correlation coefficient assessed the relationship between MDAS and OHIP-14 scores.

Multiple linear regression examined whether dental anxiety predicted OHRQoL after adjusting for age and gender, as regression residuals satisfied normality assumptions.

#### 2.8.4. Regression Diagnostics and Model Assumptions

Prior to interpretation, standard diagnostic procedures were performed to evaluate the assumptions underlying the multiple linear regression model. Normality of residuals was assessed using visual inspection of Q–Q plots and histograms, which indicated approximate normal distribution. Homoscedasticity was examined through residuals-versus-fitted values plots and showed no evidence of systematic variance patterns. Multicollinearity among independent variables was evaluated using variance inflation factors (VIFs), with all values well below commonly accepted thresholds, indicating no problematic collinearity. The presence of influential outliers was assessed using standardized residuals and Cook’s distance, and no observations were identified that would unduly influence the model estimates. These diagnostics supported the appropriateness of the regression model for the present analysis.

## 3. Results

### 3.1. Participant Characteristics

A total of 138 adult patients attending the academic dental clinic were included in the analysis. The study sample comprised both male and female participants with a broad adult age range. Descriptive characteristics of the participants are summarized in Table 1. Continuous variables are presented using measures of central tendency and dispersion appropriate to the analytical approach, while categorical variables are reported as frequencies and percentages.

### 3.2. Distribution of Dental Anxiety and OHRQoL Scores

Dental anxiety and oral health-related quality of life scores demonstrated non-normal distributions, supporting the use of non-parametric statistical methods. Overall patterns indicated variability across participants, with most individuals reporting low-to-moderate levels of dental anxiety and corresponding variation in perceived oral health impact. Detailed distributional characteristics are presented in Table 2.

### 3.3. Association Between Dental Anxiety and OHRQoL

Group-based bivariate analysis revealed statistically significant differences in OHRQoL scores across dental anxiety categories, indicating higher perceived oral health impact among participants with greater anxiety. The strength and direction of this relationship are summarized in Table 3. This finding reflects a consistent association within the study sample rather than evidence of causality.

### 3.4. Correlation Between Dental Anxiety and OHRQoL

Spearman’s rank correlation demonstrated a significant positive correlation between MDAS and OHIP-14 total scores: ρ = 0.34, *p* < 0.001.

**Figure 1:**
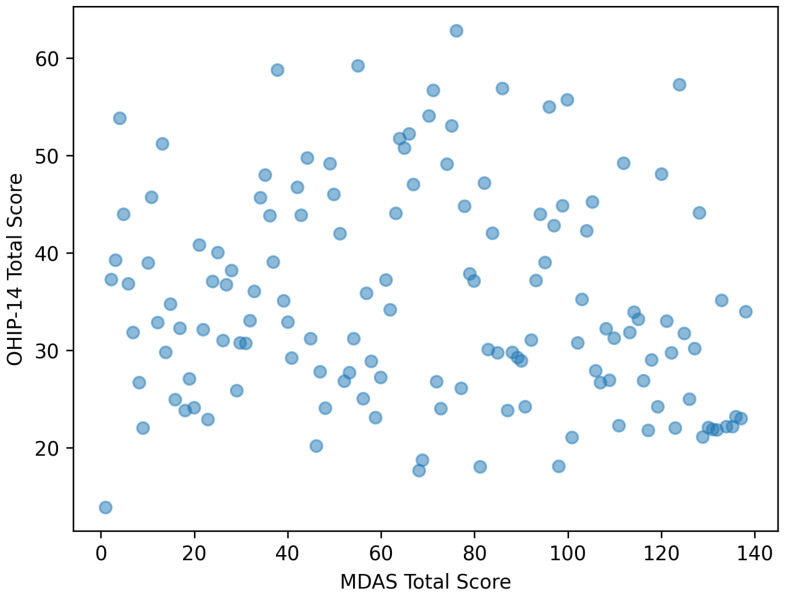
Jittered scatterplot illustrating the association between Modified Dental Anxiety Scale (MDAS) scores and OHIP-14 total scores. Each point represents an individual participant, with jitter applied to reduce overlap of discrete questionnaire scores. The figure illustrates co-occurrence patterns and does not imply causality.

This finding reflects a moderate positive association between dental anxiety and OHRQoL within the study sample, indicating that higher MDAS scores tended to co-occur with higher OHIP-14 scores. This correlation describes the strength and direction of the relationship but does not imply causation or account for potential confounding factors (Figure 1).

### 3.5. Gender Differences in Dental Anxiety and OHRQoL

Comparisons between male and female participants did not demonstrate statistically significant differences in dental anxiety or oral health-related quality of life scores. These findings indicate that no large gender-related differences were detected within the sample. However, subgroup comparisons should be interpreted cautiously, as smaller or moderate differences may not have been detectable given the sample size.

### 3.6. Factors Associated with Oral Health-Related Quality of Life

Multiple linear regression analysis was conducted as an exploratory approach to examine whether dental anxiety was independently associated with oral health-related quality of life after adjustment for age and gender.

Dental anxiety showed a statistically significant independent association with OHRQoL after adjustment for age and gender within the specified model (B = 0.69, *p* < 0.001). This indicates that higher MDAS scores were associated with higher OHIP-14 scores among the variables included in the analysis.

Age was independently associated with OHRQoL within the specified model (B = 0.11, *p* = 0.019), with higher OHIP-14 scores observed among older participants. In addition, female gender was independently associated with higher OHIP-14 scores compared with males (B = 2.14, *p* = 0.018), indicating a modest difference in perceived oral health impact within the model.

**Figure 2:**
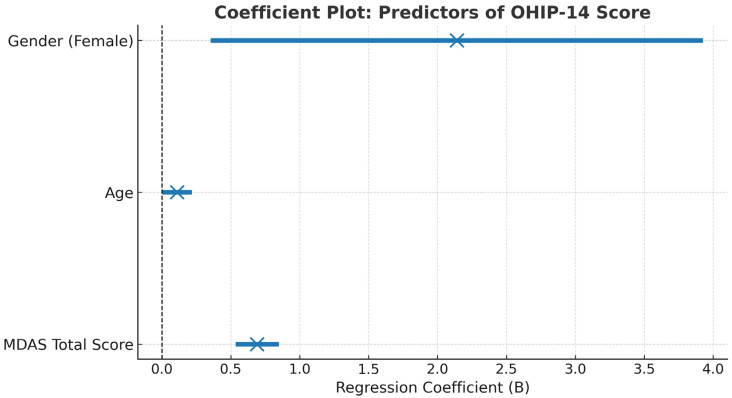
Coefficient plot showing the unstandardized regression coefficients and 95% confidence intervals for predictors of OHIP-14 total score. The × markers represent the point estimates of the regression coefficients (B) for each predictor, while the horizontal bars indicate the corresponding 95% confidence intervals. The vertical dashed line at B = 0 represents the null value, where no association is present. Dental anxiety (MDAS Total Score), age, and female gender all demonstrated statistically significant positive associations with OHIP-14 scores, indicating that higher anxiety levels, older age, and being female were associated with poorer oral health-related quality of life.

Table 4 presents the numerical regression coefficients and confidence intervals, while Figure 2 provides a visual summary of the direction and relative magnitude of associations to facilitate interpretation.

Model diagnostics were examined to assess the suitability of the multiple regression analysis. Visual inspection of residual patterns did not suggest major deviations from linearity or variance consistency, and the included variables did not show evidence of problematic interdependence. The model accounted for approximately 52% of the variability in OHIP-14 scores (R^2^ = 0.52). However, this proportion of explained variance should be interpreted cautiously, as reliance on self-reported measures and shared method variance may inflate apparent model fit in cross-sectional questionnaire-based studies.

## 4. Discussion

This study examined the association between dental anxiety and oral health-related quality of life (OHRQoL) among adults attending an academic dental clinic in the United Arab Emirates (UAE). The findings indicate that higher dental anxiety levels were associated with greater perceived oral health impact, with age and gender also showing significant associations within the analytical model employed. In this context, the present findings provide timely, region-specific evidence that contextualizes well-established international associations between dental anxiety and OHRQoL within the rapidly evolving oral healthcare landscape of the UAE.

Overall, the results highlight the substantial psychosocial burden that co-occurs with dental anxiety and reinforce its clinical relevance as a potentially modifiable factor linked to oral health perceptions. The distribution of dental anxiety levels observed in this study aligns closely with global estimates, in which most adults report low to moderate anxiety, while a smaller proportion experience high or extreme anxiety. Similar patterns have been consistently reported across diverse populations [17,18,19], suggesting that the distribution of dental anxiety may be relatively stable across cultural settings. Nevertheless, the proportion of participants reporting elevated anxiety underscores the importance of integrating anxiety-sensitive approaches into routine dental care.

An important methodological consideration when interpreting these findings is that oral health-related quality of life, as measured by the OHIP-14, is strongly influenced by underlying clinical oral conditions, including dental caries, periodontal disease, tooth loss, and malocclusion [7,20,21]. Because objective clinical indicators were not collected in the present study, the observed association between dental anxiety and OHRQoL may be partially confounded by unmeasured disease burden. Individuals with more severe oral pathology are likely to experience greater pain, functional limitations, and psychosocial distress, which may simultaneously contribute to heightened dental anxiety and poorer perceived quality of life. Consequently, the relationship observed in this study likely reflects a complex interplay between psychological and biological factors rather than a unidirectional effect of anxiety on OHRQoL.

The findings of this study should be interpreted within the context of its cross-sectional design. Although multivariable regression analysis demonstrated statistically significant associations between dental anxiety, age, gender, and OHRQoL, these relationships do not imply causation. The temporal direction of the observed associations cannot be established, and it remains plausible that poorer perceived oral health contributes to heightened dental anxiety, that anxiety influences perceived oral health impact, or that both are shaped by shared underlying factors. Accordingly, the results should be understood as evidence of association rather than confirmation of a cause–effect relationship.

OHRQoL scores in this study indicated that although many participants reported minimal functional or psychosocial impairment, nearly one-quarter experienced moderate to severe impact. This pattern is consistent with findings from previous studies in Turkey, Brazil, and Sweden, where OHRQoL tends to vary widely due to differences in dental attendance behaviors, oral hygiene practices, and cultural perceptions of oral health [22,23,24,25]. The observed moderate correlation between dental anxiety and OHRQoL is consistent with previous cross-sectional studies reporting concurrent variation between psychological distress and perceived oral health impact. Importantly, this association should be interpreted descriptively rather than causally, as the study design does not permit determination of temporal direction or exclusion of shared underlying factors. Dental anxiety and OHRQoL may reflect overlapping psychosocial constructs or be jointly influenced by unmeasured variables such as prior dental experiences, health perceptions, or coping styles, rather than one acting as a causal or confounding factor for the other [26]. Numerous studies have documented similar associations, confirming that dental anxiety not only influences care-seeking behavior but also shapes self-perceived oral health [27,28,29].

Although no statistically significant differences were observed between men and women in MDAS or OHIP-14 scores, this finding should be interpreted with caution and should not be equated with evidence of gender equality. The absence of statistical significance indicates that no large gender differences were detected within the present sample; however, smaller or moderate differences may have remained undetected due to limited statistical power, particularly in subgroup analyses. This contrasts with earlier literature reporting higher dental anxiety levels among females [30,31]. Several factors may explain this discrepancy. Cultural shifts in attitudes toward dental care and increasing oral health awareness in the UAE may have attenuated historically reported gender differences [32]. In addition, the relatively balanced gender distribution and similar dental attendance patterns within the study population may have reduced observable variation. Differences in sample composition compared with prior studies, many of which included populations with more pronounced age or socio-demographic skewing [33,34] may also contribute. Notably, despite the lack of statistically significant bivariate gender differences, regression analysis demonstrated an independent association between female gender and poorer OHRQoL, suggesting that gender-related differences in perceived oral health impact may exist even when mean score differences are not statistically significant. This finding aligns with broader evidence indicating that women tend to report higher subjective symptom burden and greater sensitivity to functional and psychosocial health impacts [33,34].

The graded differences in OHRQoL across anxiety categories indicate a consistent, monotonic association between higher anxiety levels and poorer perceived oral health impact, supporting the conceptual framework in which higher anxiety disrupts dental care utilization, increases avoidance of treatment, and exacerbates oral discomfort and psychosocial stress [5,10]. These findings are in agreement with prior works, which consistently show that highly anxious individuals exhibit poorer oral health habits, increased caries and periodontal burden, and reduced dental satisfaction. The significant differences between low, moderate, and high anxiety groups also mirror results from large-scale epidemiological studies in Europe, thereby reinforcing the external validity of the present findings [35,36,37,38].

Multivariable regression analysis demonstrated that dental anxiety was independently associated with oral health-related quality of life after adjustment for age and gender. However, this model was intentionally limited to a small number of covariates and does not represent a comprehensive confounding structure. Accordingly, the observed association should be interpreted within the context of the variables included, rather than as evidence of a dominant or causal role of dental anxiety relative to other unmeasured factors. This pattern mirrors the conclusions of McGrath and Bedi and recent multinational analyses, which position dental anxiety as one of the most consistent psychosocial determinants of poor oral health outcomes [39]. The finding that age predicted worse OHRQoL aligns with prior studies showing that older adults may face more cumulative dental issues, greater treatment needs, and heightened sensitivity to oral discomfort [40]. The observed gender effect is also consistent with psychological literature suggesting that females tend to express greater awareness of emotional and social impacts related to their health, although biological and hormonal influences may also contribute [41,42]. Some inconsistencies with the literature, such as the absence of raw gender differences in anxiety scores, may reflect unique socio-cultural characteristics of the UAE population, including increased dental awareness, improved access to care, and shifting perceptions of dental treatment across genders. Additionally, variations in sample composition, clinic setting, and health-seeking behavior may explain why certain studies report stronger gender differences while others such as the present study do not.

The proportion of explained variance should be interpreted cautiously, as shared method variance and reliance on self-reported measures may inflate apparent model fit in cross-sectional questionnaire-based studies.

The regression model was intentionally parsimonious and limited to variables consistently available across participants. While important potential confounders such as socioeconomic status, oral hygiene practices, psychological comorbidities, and patterns of dental service utilization were not included, their omission reflects data availability rather than analytical oversight. Consequently, the observed relationships should be interpreted as adjusted but not fully controlled associations, and residual confounding cannot be excluded. Future studies using longitudinal designs, broader variable inclusion, and multi-center sampling are required to clarify causal pathways and disentangle these interrelated influences.

While this study provides valuable insights into the association between dental anxiety and oral health-related quality of life, several limitations should be considered when interpreting the findings. A major methodological limitation is the absence of objective clinical oral health indicators, such as caries experience, periodontal status, tooth loss, or malocclusion. Because OHIP-14 scores are directly influenced by the presence and severity of oral disease, the observed associations between dental anxiety and OHRQoL may be partially attributable to unmeasured clinical factors. This limits the ability to disentangle the independent psychological contribution of dental anxiety from the direct effects of underlying oral pathology on daily functioning and well-being. Consequently, the relationship observed in this study should be interpreted as reflecting a complex interplay between psychological and biological influences rather than a unidirectional effect.

A further methodological consideration is the substantial deviation between the planned and achieved sample size. Although statistically significant associations were observed, the reduced sample size limits the precision of effect estimates and the ability to detect smaller or more nuanced associations. Accordingly, the findings should be interpreted as exploratory and hypothesis-generating rather than confirmatory, and the absence of statistically significant differences in certain subgroup analyses should not be interpreted as evidence of equivalence.

In addition, participants were recruited using a non-probabilistic sampling strategy from a single academic dental institution. This approach may limit external validity, as individuals seeking care in an academic clinical setting may differ from the general population with respect to oral health awareness, healthcare-seeking behavior, and psychosocial characteristics. Consequently, the generalizability of the findings to the wider adult population in the United Arab Emirates should be interpreted with caution.

Although multivariable regression analysis was used to adjust for age and gender, other potentially relevant determinants of oral health-related quality of life—including clinical disease burden, socioeconomic status, oral hygiene practices, dental attendance patterns, previous dental experiences, and psychological comorbidities—were not included in the analytical models. The omission of these variables reflects data availability rather than analytical oversight and leaves the possibility of residual confounding that may influence the observed associations. Therefore, the regression findings should be understood as adjusted associations within a limited covariate framework rather than as comprehensive estimates of the relative importance of determinants of OHRQoL.

Finally, the cross-sectional design precludes any inference regarding temporal direction or causality. Although consistent associations were observed between dental anxiety and oral health-related quality of life, these findings should not be interpreted as evidence of causal or predictive relationships, nor should they be extrapolated to future outcomes. Future longitudinal, multi-center studies employing probabilistic sampling strategies, objective clinical assessments, and broader covariate adjustment are required to confirm these findings, clarify causal pathways, and strengthen generalizability.

## 5. Conclusions

This cross-sectional study examined the association between dental anxiety and oral health-related quality of life among adult patients attending an academic dental clinic in the United Arab Emirates. Within the limitations of a modest, non-probabilistic sample, higher dental anxiety levels were consistently associated with poorer perceived oral health impact. Age and gender also showed statistically significant associations with oral health-related quality of life within the analytical model employed.

These findings should be interpreted strictly as evidence of association rather than causation. The cross-sectional design does not permit inference regarding temporal direction, causal mechanisms, or prediction of future outcomes, and the regression analysis was limited to a small set of available covariates. Accordingly, the results should not be interpreted as identifying primary or dominant predictors of oral health-related quality of life, nor should they be generalized to the wider adult population of the United Arab Emirates.

Despite these limitations, the study contributes updated, region-specific evidence to a limited Middle Eastern literature and highlights dental anxiety as a relevant psychosocial factor that co-occurs with poorer self-reported oral health outcomes among dental clinic attendees. Future research using longitudinal designs, probabilistic sampling, objective clinical indicators, and broader contextual variables is required to clarify causal pathways and inform evidence-based clinical or public health strategies.

Future studies should integrate standardized clinical oral examinations with psychosocial assessments in order to disentangle the relative contributions of disease burden and psychological distress to oral health-related quality of life.

## Figures and Tables

**Table 1:** Demographic Characteristics of the Participants (*n* = 138).

Characteristic		Value
Age (years)		Mean ± SD: 34.9 ± 10.9
Median (IQR): 32.0 (26.0–42.0)		
Gender	Male: 76 (55.1%)	
Female: 62 (44.9%)		

Continuous variables are presented as mean ± standard deviation and median (interquartile range) to ensure consistency with the nonparametric analytical approach used.

**Table 2:** Descriptive Statistics for MDAS and OHIP Scores.

Statistic	MDAS Total Score	OHIP Total Score
N	138	138
Mean	10.54	14.22
Std. Deviation	5.03	11.64
Minimum	5.00	0.00
25th Percentile	7.00	4.25
Median (50th Pctl)	10.00	12.00
75th Percentile	13.25	23.00
Maximum	25.00	55.00

**Table 3:** Comparison of OHIP-14 scores Across Dental Anxiety Categories Using the Kruskal–Wallis Test and Bonferroni-Adjusted Post Hoc Analysis.

Anxiety Category	n	Mean OHIP-14	SD	Median (IQR)	Comparison	Adjusted *p*-Value	Significance
Low Anxiety	68	12.77	6.92	6.5 (2.0–19.25)	Low vs. Moderate	0.022	*
Moderate Anxiety	53	16.72	7.18	14 (7.0–18.0)	Low vs. High	<0.001	***
High Anxiety	17	24.75	7.47	24 (18.0–33.0)	Moderate vs. High	<0.001	***

Kruskal–Wallis Test: H = 17.29, *p* < 0.001. Significance Code: * = *p* < 0.05, *** = *p* < 0.001.

**Table 4:** Multiple Linear Regression Predicting OHIP-14 Total Score.

Variable	B (Unstandardized Coef.)	SE	t-Value	*p*-Value	95% CI (Lower)	95% CI (Upper)
Constant	5.91	1.91	3.09	0.002	2.12	9.69
MDAS Total Score	0.69	0.07	9.90	<0.001	0.55	0.83
Age	0.11	0.05	2.38	0.019	0.02	0.20
Gender (1 = Female)	2.14	0.89	2.39	0.018	0.37	3.91

Model diagnostics: R^2^ = 0.52; adjusted R^2^ = 0.51; all VIFs < 2.0.

## Data Availability

The data supporting the findings of this study are available from the corresponding author upon reasonable request. The dataset contains individual-level research data that cannot be fully deposited in a public repository due to ethical and institutional restrictions, including participant confidentiality and data protection requirements stipulated by our ethics approval.
